# Supplementary material for: Influence of Fraction Particle Size of Pure Straw and Blends of Straw with Calcium Carbonate or Cassava Starch on Pelletising Process and Pellet
Source: Materials (Basel). 2020 Oct 16;13(20):4623. doi: 10.3390/ma13204623 (PMC7602947; doi:10.3390/ma13204623)
Supplement: Supplementary file 1 [file materials-13-04623-s001.pdf]

materials

MDPI

Supplementary Information

Influence of Fraction Particle Size of Pure Straw and Blends of Straw with Calcium Carbonate or Cassava Starch on Pelletising Process and Pellet

Table S1. Descriptive parameters of process pelletising and pellets properties.

Parameter	Formula
Fraction particle size, geometric mean of particle size on the *i*-th sieve (mm)	$x = x_{si} = \sqrt{x_i x_{i-1}}$
Maximum agglomeration pressure (MPa)	$p_m = \frac{4F_m}{\pi d_m^2}$
Specific compaction work (kJ·kg⁻¹)	$L_s = \int_0^{s_m} F\,dx/[m_s(1 - MC/100)]$
Specific work for pellet shift in the die (kJ·kg⁻¹)	$L_v = \int_{s_m}^{(s_e - s_m)} F\,dx/[m_s(1 - MC/100)]$
DM single pellet density (kg·m⁻³)	$\rho_p = \frac{32m(1 - MC/100)}{\pi(d_1 + d_2)^2(l_{p1} + l_{p2})}$
Specific compression work to pellet cracking (mJ·mm⁻²)	$E_j = \frac{1}{S}\int_0^{\Delta l} F_{cm} dx$
Elasticity modulus for pellet compression (MPa)	$E = \frac{F_c d}{S \Delta l}$
Maximum tensile strength during pellet cracking (MPa)	$\sigma_c = \frac{2F_{cm}}{\pi d l_p}$
Water absorption (g H_2O·g⁻¹ DM)	$k = (m_{s1} - m_{s0})/m_{s0}(1 - MC/100)$

where x and x_{si}, geometric mean of particle size on the *i*-th sieve; x_i, holes diagonal of *i*-th sieve; $x_{(i-1)}$, diagonal of sieve hole, which is above the *i*-th sieve; p_{max}, maximum compaction pressure; F_{max}, maximum compaction force d_m, die hole diameter; L_s and L_v, DM specific works of compaction and pellet shift in the die; s_m and s_e, piston displacement to achieve a proper maximum compaction pressure and end piston position after pellet displacement in the die ($v_p = s_e - s_m$); v_p, pellet shift in the die; F, compaction force; m_s, dose mass; MC, material moisture in reference to wet substance; ρ_p, DM single pellet density; m, pellet mass; d_1 and d_2, pellet diameters in two perpendicular directions; l_{p1} and l_{p2}, pellet lengths in two perpendicular directions; E_j, energy of compression work until pellet cracking; F_c, compressive force; S, pellet surface on which the load works; E, elasticity modulus during pellet compression; F_{cs}, pellet tensile strength within the range of elasticity; d, pellet diameter before loading; Δl, pellet deformation under load (mm); σ, maximum tensile strength during pellet cracking; l_p, pellet length; k, water absorption by pellets; m_{s1}, weight of pellet sample after soaking in water; m_{s0}, weight of pellet sample before soaking in water.

Table S2. Correlation matrix for type of additive, fraction particle size x, pellet dimeter d, DM single pellet density ρ_p, elasticity modulus for pellet compression E, specific pellet compression work E_j, tensile strength σ_c, piston displacement at maximum agglomeration pressure s_m, pellet shift in the die v_p, specific compaction work L_s, specific work for pellet shift in the die L_v, water absorption by crushed pellets k, and water absorption by uncrushed pellets k_u.

Parameter	Additive	x	d	ρ_p	E	E_j	σ_c	s_m	v_p	L_s	L_v	k	k_u
Additive	1.000												
x	0.000	1.000											
d	−0.416[a]	0.194	1.000										
ρ_p	0.284[a]	0.173	−0.005	1.000									
E	0.676[a]	−0.035	−0.336[a]	0.456[a]	1.000								
E_j	0.390[a]	0.100	−0.198	0.453[a]	0.506[a]	1.000							
σ_c	0.580[a]	0.140	−0.329[a]	0.661[a]	0.626[a]	0.750[a]	1.000						
s_m	0.015	0.826[a]	0.180	0.181	0.100	0.198	0.196	1.000					
v_p	0.012	−0.182	−0.017	−0.068	−0.064	−0.072	−0.050	−0.532[a]	1.000				
L_s	0.183	0.565[a]	0.063	0.098	−0.029	0.172	0.236	0.495[a]	−0.414[a]	1.000			
L_v	−0.132	0.181	0.070	−0.100	−0.221	−0.130	−0.094	−0.244	0.705[a]	0.161	1.000		
k	0.365[a]	0.150	−0.172	−0.287[a]	0.116	−0.278[a]	−0.247	0.112	−0.010	0.124	0.004	1.000	
k_u	−0.433[a]	0.390[a]	0.215	−0.173	−0.424[a]	−0.091	−0.132	0.243	−0.010	0.176	0.237	−0.260[a]	1.000

[a] statistically significant at *p*-value = 0.05

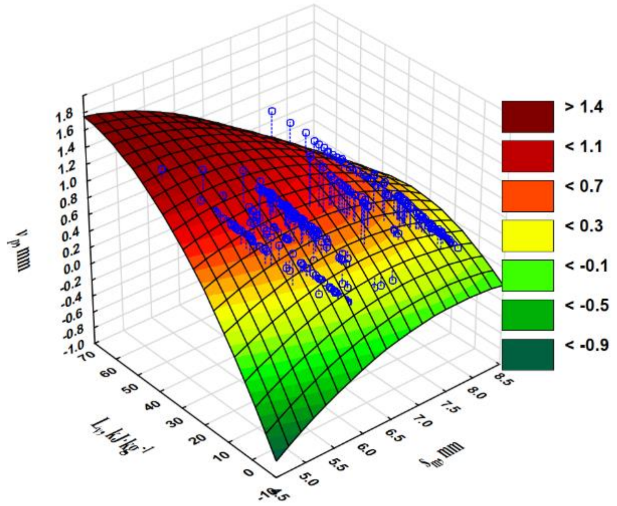

Figure S1. Pellet shift in die v_p vs. piston displacement s_m and the specific work of the pellet shift in the die L_v.

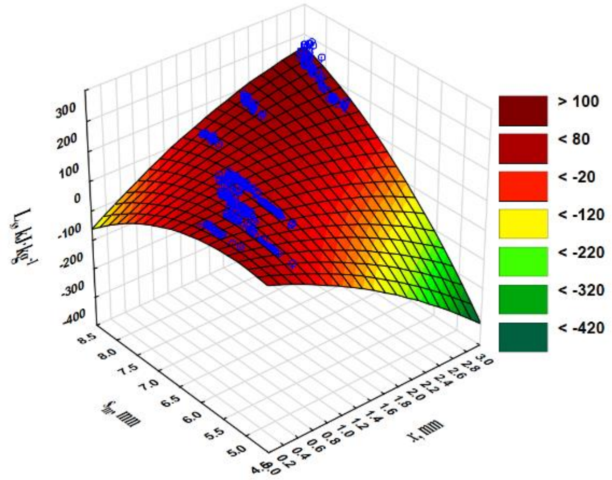

Figure S2. Pellet shift in die v_p vs. piston displacement s_m and the specific work of the pellet shift in the die L_v.
